# *O*-GlcNAcylation on Rab3A attenuates its effects on mitochondrial oxidative phosphorylation and metastasis in hepatocellular carcinoma

**DOI:** 10.1038/s41419-018-0961-7

**Published:** 2018-09-20

**Authors:** Weicheng Wu, Xixi Zheng, Jing Wang, Tianxiao Yang, Wenjuan Dai, Shushu Song, Lan Fang, Yilin Wang, Jianxin Gu

**Affiliations:** 1 Department of Biochemistry and Molecular Biology, School of Basic Medical Sciences, Fudan University, Shanghai, China; 2 Key Laboratory of Glycoconjugate Research Ministry of Health, School of Basic Medical Sciences, Fudan University, Shanghai, China; 3 Shanghai Tenth People’s Hospital of Tongji University, School of Medicine and School of Life Science and Technology, Tongji University, Shanghai, China; 4 Department of Hepatic Surgery, Fudan University Shanghai Cancer Center, Shanghai Medical College, Fudan University, Shanghai, 200032 China; 5 Department of Oncology, Shanghai Medical College, Fudan University, Shanghai, 200032 China

## Abstract

Rab3A is a small Ras-like GTPase critical for membrane traffic. Although the functions of Rab3A have been reported in several cancers, the roles of Rab3A in hepatocellular carcinoma (HCC) have never been determined. To investigate the potential roles of Rab3A in HCC progression, we first determined Rab3A levels in HCC tissues and observed upregulated mRNA and protein levels of Rab3A in most tumor tissues. However, in vitro data showed that decreasing Rab3A in most HCC cell lines conferred no significant effects and overexpressing Rab3A in PLC/PRF/5 cells even inhibited migration and invasion. Meanwhile, the upregulation of Rab3A in HCC patients did not correlate with metastasis or overall survival of HCC patients. These contradict data suggested that Rab3A might act as metastatic suppressor and its effects might be attenuated in most HCC cells. Further experiments revealed that *O*-GlcNAcylation on Rab3A was key for attenuating Rab3A-mediated effects by regulating its GTP-binding activity, and verified the effects of Rab3A and its aberrant *O*-GlcNAcylation on HCC metastasis in vitro and in vivo. We also found that Rab3A and its *O*-GlcNAcylation had opposite roles in mitochondria oxidative phosphorylation (mtOXPHOS), and their functions on HCC metastasis were partially depended on their effects on metabolic reprogramming.

## Background

In most cancers, cancer cells reprogram their energy metabolism^1^ and adopt aerobic glycolysis rather than oxidative phosphorylation (OXPHOS) to supply energy, which is known as the “Warburg effect^2^.” This process can be driven by oncogenic pathways involving different oncogenes^1,3^. The inactivation or downregulation of some tumor suppressors confers similar effects^1,4^. During metabolism reprogramming, enhanced glucose uptake and hexosamine biosynthetic pathway flux increases cytoplasmic UDP-GlcNAc levels and elevates cellular *O*-GlcNAcylation^5^. *O*-linked β-*N*-acetylglucosamine (*O*-GlcNAc) is the covalent addition of a GlcNAc sugar moiety to hydroxyl groups of serine/threonine residues of proteins^6^. *O*-GlcNAc transferase (OGT) transfers the GlcNAc moiety from the high-energy donor UDP-GlcNAc to substrate proteins and *O*-GlcNAcase (OGA) hydrolyzes *O*-GlcNAc from the proteins^7^. Aberrantly elevated *O*-GlcNAcylation is observed in nearly all human malignancies examined^8^ and modulates metabolic reprogramming in return^5^. *O*-GlcNAcylation not only regulates the activities of different glycolytic enzymes^9,^ but also controls the oncogenic signaling pathways and the stabilization of some tumor suppressors in cancer^10–12^.

Rab proteins belong to the small Ras-like GTPase superfamily, and function in vesicle trafficking, membrane targeting, and fusion^13^. Rab protein activity is controlled by cycling between the active GTP-bound and inactive GDP-bound forms^14^. Altered GTP/GDP-binding of Rabs by mutations or dysregulation in Rabs levels dampens the efficiency and specificity in membrane traffic, which is implicated in different diseases including cancers^15^. Among the Rab superfamily, Rab3A is the key regulator in transporting cellular products into secretory vesicles and lysosomes^16^. Aberrantly upregulated Rab3A was observed in human insulinoma, breast cancer, and tumors derived from the neural system^17–20^. Some functional partners of Rab3A such as Rab27A, Spire-1, and β-APP are also associated with the malignant characters in cancers^21–23^. However, the role of Rab3A in hepatocellular carcinoma (HCC) has never been determined.

In this study, we observed the upregulation of Rab3A in HCC. However, different from its oncogenic roles in other neoplasms, Rab3A suppresses metastasis by enhancing OXPHOS in HCC, which is attenuated by the *O*-GlcNAcylation on Rab3A. Furthermore, due to the common hyper-*O*-GlcNAcylation in HCC tumor cells, upregulated Rab3A malfunctions in most HCC patients.

## Material and methods

### Patients and samples

Usage of human pathological tissues and clinical data was approved by the ethics committee in Shanghai Cancer Center of Fudan University (Shanghai, China). All patients’ written consent conformed to the ethical guidelines of the Helsinki Declaration. A total of 180 patients with primary HCC resected between 2010 and 2012 in Department of Hepatic Surgery, Shanghai Cancer Center of Fudan University (Shanghai, China) were collected. None of the patients had received pre-operative therapy. Clinical stages of tumor were determined according to the TNM classification system of International Union against Cancer. Follow-up was finished until 9 December 2016. These patients were followed every 3 months. The median follow-up was 33.3 months (ranging from 0.8 to 60.4 months). Among all the primary tumor specimens, 16 of them were used for Western blotting (WB) and quantitative real-time PCR (qPCR) assays. For *O*-GlcNAcylation detection, three pairs of fresh tumor tissues and adjacent normal tissues were resected from HCC patients (all male, age from 40 to 60 years, without pre-operative therapy, from Department of Hepatic Surgery, Shanghai Cancer Center of Fudan University Shanghai, China) from May 2018 to June 2018.

### Cell culture

All the HCC cell lines and 293T cells were obtained from Cell Bank of Type Culture Collection of Chinese Academy of Sciences (Shanghai, China) and cultured in Dulbecco’s minimum essential medium (DMEM) supplemented with 10% fetal bovine serum (FBS) at 37 °C in a humidified atmosphere containing 5% CO_2_. The FBS and the DMEM culture media were purchased from Sigma (St. Louis, MO, USA).

### Animal models

All the animal experiments were approved by the research medical ethics committee of Fudan University (Shanghai, China) and were performed in accordance with the approved guidelines. The nude mice were purchased from Shanghai Laboratory Animal Center of Chinese Academy Sciences (Shanghai, China) and were housed in individual ventilated cages. All of the mice were randomly grouped (*n* = 6 in each group). PLC/PRF/5-luc cell suspension in phosphate-buffered saline (PBS) (5 × 10^5^/mouse) with indicated treatments was injected intravenously into the lateral tail vein of 6-week-old male BALB/c-nu/nu mice. Bioluminescent imaging was performed every week after injection with IVIS200 (Xenogen, Caliper, CA). Every image was fetched 10 min after the intraperitoneal injection of luciferin (3 mg/mouse) (Promega, WI, USA). The intensity of signals was quantified using region of interest analysis.

### Immunohistochemistry

Immunohistochemistry (IHC) analysis was applied on tissue microarray using Dako REAL EnVision Detection System (Dako, Denmark) according to the manufacturer’s instruction. Anti-Rab3A and anti-OGT antibodies were used for staining. Hematoxylin was used for counterstaining. Immunohistochemical scoring was determined as previous described^24^. The staining intensity was scored as 0 for negative, 1 for weak, 2 for moderate weak, 3 for moderate strong, and 4 for strong. The score for the stained area was set as 0 for 0–33%, 1 for 33–66%, and 2 for 66–100%. The final staining score was obtained by multiplying staining intensity score with staining area score and the results are a series number ranging from 0 to 8.

### Plasmid construction

The cDNA encoding Rab3A or OGT was obtained by PCR and was inserted into the pCMV-Flag vector (Sigma). The sequences of short hairpin RNA inserted in pENTR/U6 vector (Thermo, USA) are listed as follows. Rab3A: 5′- CACCGCATCGACTTCAAGGTCAAGACGAATCTTGACCTTGAAGTCGATGC-3′, OGT: 5′-CACCGCTTGCAATTCATCACTTTGACGAATCAAAGTGATGAATTGCAAGC-3′, COX8A: 5′- CACCGCCGCGCGCCAAGATCCATTCCGAAGAATGGATCTTGGCGCGCGGC-3′. Transfections were performed with Lipofectamine 3000 (Life Technologies, CA, USA), according to the manufacturer’s instructions. Stable cell lines were generated with G418 (200 μg/mL) in the medium.

### Quantitative real-time PCR

Total RNA samples were purified using TRIzol (Invitrogen, Carlsbad, CA, USA) and then transcribed to cDNA using PrimeScript RT reagent Kit (Takara, Tokyo, Japan). Real-time PCR was performed with the cDNA production using SYBR Premix Ex Taq (Takara) on ABI StepOne Plus (Applied Biosystems, USA). GAPDH was used as an internal control. Relative expression level was computed using 2^−ΔΔCt^ method. The primer sequences used are listed below.

All the primers used in this study were listed below: Rab3A forward, 5′-GAGTCCTCGGATCAGAACTTCG-3′; Rab3A reverse, 5′-TGTCGTTGCGATAGATGGTCT-3′; OGT forward, 5′-TCCTGATTTGTACTGTGTTCGC-3′; OGT reverse, 5′-AAGCTACTGCAAAGTTCGGTT-3′; COX8A forward, 5′-GCCAAGATCCATTCGTTGCC-3′; COX8A reverse, 5′-CTCTGGCCTCCTGTAGGTCT-3′; COX7C forward, 5′-GGTCCGTAGGAGCCACTATGA-3′; COX7C reverse, 5′-GTGTCTTACTACAAGGAAGGGTG-3′; COX6A1 forward, 5′-AGTTGGTGTGTCCTCGGTTTC-3′; COX6A1 reverse, 5′-GTGAGAGTCTTCCACATGCGA-3′; COX5B forward, 5′-ATGGCTTCAAGGTTACTTCGC-3′; COX5B reverse, 5′-CCCTTTGGGGCCAGTACATT-3′; COX5A forward, 5′-ATCCAGTCAGTTCGCTGCTAT-3′; COX5A reverse, 5′-CCAGGCATCTATATCTGGCTTG-3′; COX4I1 forward, 5′-CAGGGTATTTAGCCTAGTTGGC-3′; COX4I1 reverse, 5′-GCCGATCCATATAAGCTGGGA-3’; GAPDH forward, 5′-GAGTCAACGGATTTGGTCGT-3′; and GAPDH reverse, 5′-TTGATTTTGGAGGGATCTCG-3′.

### Transwell assay

Cell transwell migration and invasion were assessed at 8 μm transwell filters (Millipore, Billerica, MA, USA) in a 12-well plate. For invasion assays, the bottom of transwell chamber was coated with BD Matrigel Basement Membrane Matrix (BD Biosciences, San Diego, CA, USA). Tumor cells were added into the upper chamber containing serum-free DMEM with 10% bovine serum albumin and the lower chamber was filled with DMEM supplemented with 10% FBS. For migration assays, DMEM with 10% FBS was added into both chambers. Cell migration or invasion was determined 48 h later. Cells on the upper side of the chamber were removed from the surface of the membrane by scrubbing and cells on the lower surface of the membrane were fixed with 4% paraformaldehyde and stained with 0.1% crystal violet. The number of migrated or invasive cells was counted in five randomly selected microscopic fields of each filter.

### Oxygen consumption rate measurement

The mitochondrial respiratory capacity was determined using XF Cell Mito Stress Test Kit (Agilent Technologies). Cells seeded in the XF Cell Culture Microplate were incubated for 24 h. Next day, cells were incubated with the base medium containing 2 mM l-glutamine, 1 mM sodium pyruvate, and 10 mM glucose for 1 h before assay. The oxygen consumption rate (OCR) was measured by XF96 extracellular flux analyzer (Agilent Technologies) with sequential injection of 1 μM oligomycin A, 0.5 μM FCCP, and 0.5 μM rotenone/antimycin A. Each point in the traces represents the average measurement from six different wells.

### Lactate assay

Lactate concentrations were detected according to the manufacturer’s protocol (Sigma). Briefly, cells were homogenized in four volumes of lactate assay buffer and centrifuged at 13,000 × *g* for 10 min to remove insoluble material. A master reaction mix containing 20 μl sample solution, 26 μl lactate assay buffer, 2 μl lactate enzyme mix, and 2 μl lactate probe was added, and reactions were incubated at room temperature for 30 min. Sample absorbance was measured at 570 nm (A570) on a microplate reader. A total of 10 μl of 100 nmol/μl lactate standard was diluted with 990 μl lactate assay buffer to generate a 1 nmol/μl standard solution. The volumes of the 1 nmol/μl lactate standard solution used to generate the standard curve were 0, 2, 4, 6, 8, and 10 μl.

### ROS-Glo H_2_O_2_ assay

ROS-Glo H_2_O_2_ assays were performed according to the manufacturer’s protocol (Promega). Cells were incubated with H_2_O_2_ substrate solution for 6 h. The media was then incubated with the ROS-Glo detection solution containing d-Cysteine and the signal enhancer solution for 20 min at room temperature. Luminescence intensities from the mixture were measured by the GloMaxMulti Detection System (Promega).

### Mitochondrial superoxide detection

Mitochondrial superoxide was detected using the fluorescent MitoSox probe (Invitrogen). Cells were incubated with 2 μM MitoSox Red for 30 min and the fluorescence assessed using a FACS Calibur flow cytometer (BD Biosciences, NJ, USA). Thresholds were adjusted by using non-stained and stained cells for MitoSOX fluorescence.

### Immunoprecipitation and Western blotting

Immunoprecipitation (IP) was performed using Protein G IP Kit (Roche, Switzerland) according to the manufacturer’s instruction. For *O*-GlcNAcylation detection on tissues, 300 µg samples and 2 µg antibody were used in each IP test. For WB analysis, immunoprecipitated beads, cell pellets, or HCC tissues were solubilized in RIPA buffer (Beyotime Institute of Biotechnology, China) at 4 °C. Protein concentration was determined using the Quick Start™ Bradford protein assay kit (Bio-Rad, USA) and 10 μg of total protein extracts was loaded in 10% SDS-polyacrylamide gel electrophoresis (PAGE), electrophoresed, and transferred to 0.45 μm polyvinylidene difluoride membranes (Millipore). The membranes were incubated with indicated primary antibodies followed by the incubation with horseradish peroxidase (HRP)-conjugated secondary antibodies (Jackson ImmunoResearch, UK). Blotted proteins were visualized using the Immobilon™ Western Chemiluminescence HRP substrate kit (Millipore). Images were obtained from the ImageQuant™ LAS-4000 (Amersham Biosciences, GE, USA) and quantified using the ImageQuant™ TL software (version 7.0, Amersham Biosciences). Antibodies against Rab3A (Proteintech, USA) and myc-tag (Invitrogen) were used for both IP and WB analysis, and antibodies against OGT, OGA, acetylation, phosphor-Thr, phosphor-Ser, *O*-GlcNAc, GAPDH (Abcam, USA), and several Cytochrome C oxidases (COXs) (Proteintech) were used in WB analysis.

### GST pull-down

Full-length Rab3A cDNA was cloned into pGEX4T-1 vector and transformed into *Escherichia coli*. Soluble GST-Rab3A was purified on GST-beads from *E. coli* 8 h after transformation and OGT protein was synthesized in TNT reaction with [35 S]Methionine. After confirming the positive expression of GST-Rab3A and OGT via WB analysis, further pull-down assay was performed with MagneGST pull-down system (Promega) according to the manufacturer’s instruction. Pull-down result was analyzed with SDS-PAGE and autoradiography.

### GTP-binding assay

Cells were rinsed in ice-cold PBS and collected in GTP-binding buffer (20 mm Tris-HCl pH 7.5, 5 mm MgCl_2_, 2 mm phenylmethylsulfonyl fluoride, 150 mm NaCl, 0.1% Triton X-100, 0.025 mm PUGNAc, and 1:1000 diluted protease and phosphase inhibitor mixture). Samples were sonicated and clarified by centrifugation. The supernatant was collected and pre-cleared using the control agarose resin, and then incubated with 100 μl of GTP-agarose beads (Sigma-Aldrich) in a total of 500 μl of GTP-binding buffer overnight at 4 °C. The beads were washed seven times and bound protein was eluted from the beads by boiling them in reducing SDS-PAGE buffer. Rab3A that was pulled-down by the GTP-agarose beads was quantified by WB analysis.

### Statistical analysis

All analyses were performed with SPSS 13.0 (Chicago, IL, USA) and R software. Results were presented as means ± SD with at least three replicates for each sample. Optimal cutoff value of Rab3A expression was determined by receiver operating characteristic (ROC) curve analysis. Pearson’s *χ*^2^-test was used to identify the correlation between Rab3A expression and other factors. Survival probability was determined by Kaplan–Meier curve and the differences between groups were assessed by Log-rank test. Differences between groups were determined with Student’s *t*-test. Statistical significance was set at two-tails *p* < 0.05.

## Results

### Rab3A is upregulated in HCC

**Fig. 1:**
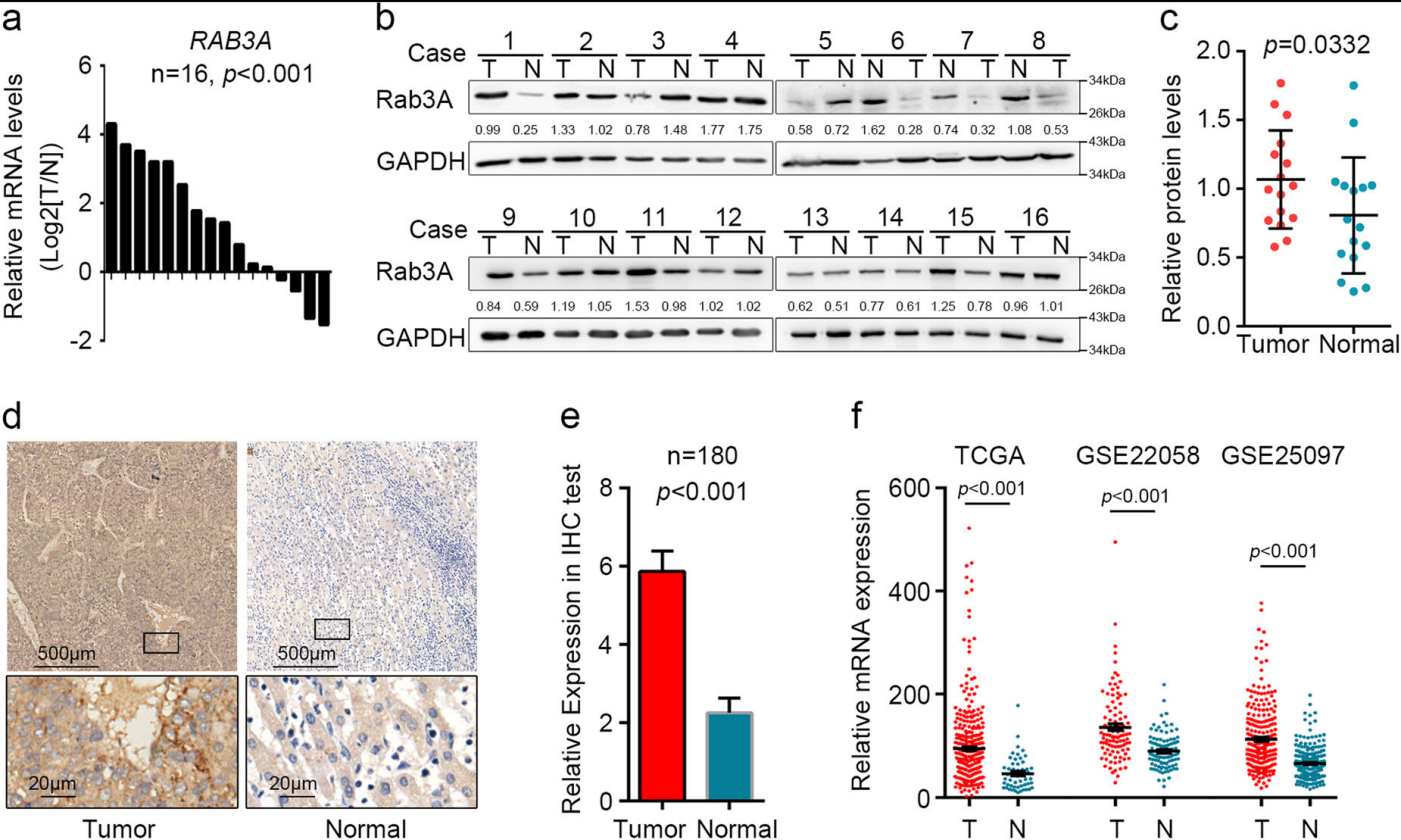
Rab3A is upregulated in HCC tumor tissues. **a** Relative mRNA expression of *RAB3A* in 16 pairs of HCC tumor tissues and adjacent non-tumor tissues was determined by real-time PCR. **b** Western blot analysis was used to determine protein levels of Rab3A in 16 pairs of HCC tissues. T, HCC tumor tissue; N, adjacent non-tumor tissue. **c** Statistical data of Western blot analysis on HCC tumor tissues and adjacent non-tumor tissues. **d** Representative IHC staining of tumor tissues and adjacent non-tumor tissues in HCC. Regional magnification images were showed below. **e** Statistical data of the IHC staining scores. **f** Relative mRNA expression of *RAB3A* in HCC tissues from GSE22058, GSE25097, and TCGA-LIHC datasets. ***p < 0.001

Higher levels of Rab3A in tumor tissues compared with adjacent normal tissues were determined by qPCR and WB analysis in 16 pairs of HCC tissue samples (*p* < 0.001) (Fig. 1a–c and Additional file 1: Figure S1a-b). Further IHC assay on 180 pairs of HCC samples also revealed the significant upregulation of Rab3A in HCC tumor tissues (*p* < 0.001) (Fig. 1d–e). Furthermore, similar results were observed in published HCC datasets TCGA-LIHC, GSE22058^25^, and GSE25097^26^ (Fig. 1f). Meanwhile, L02 normal liver cell line expressed less Rab3A than different HCC cell lines as well (Additional file 1: Figure S1c-d). These data indicated that Rab3A is upregulated in HCC.

### Rab3A malfunctions in regulating metastasis of most HCC cells and HCC patients

**Fig. 2:**
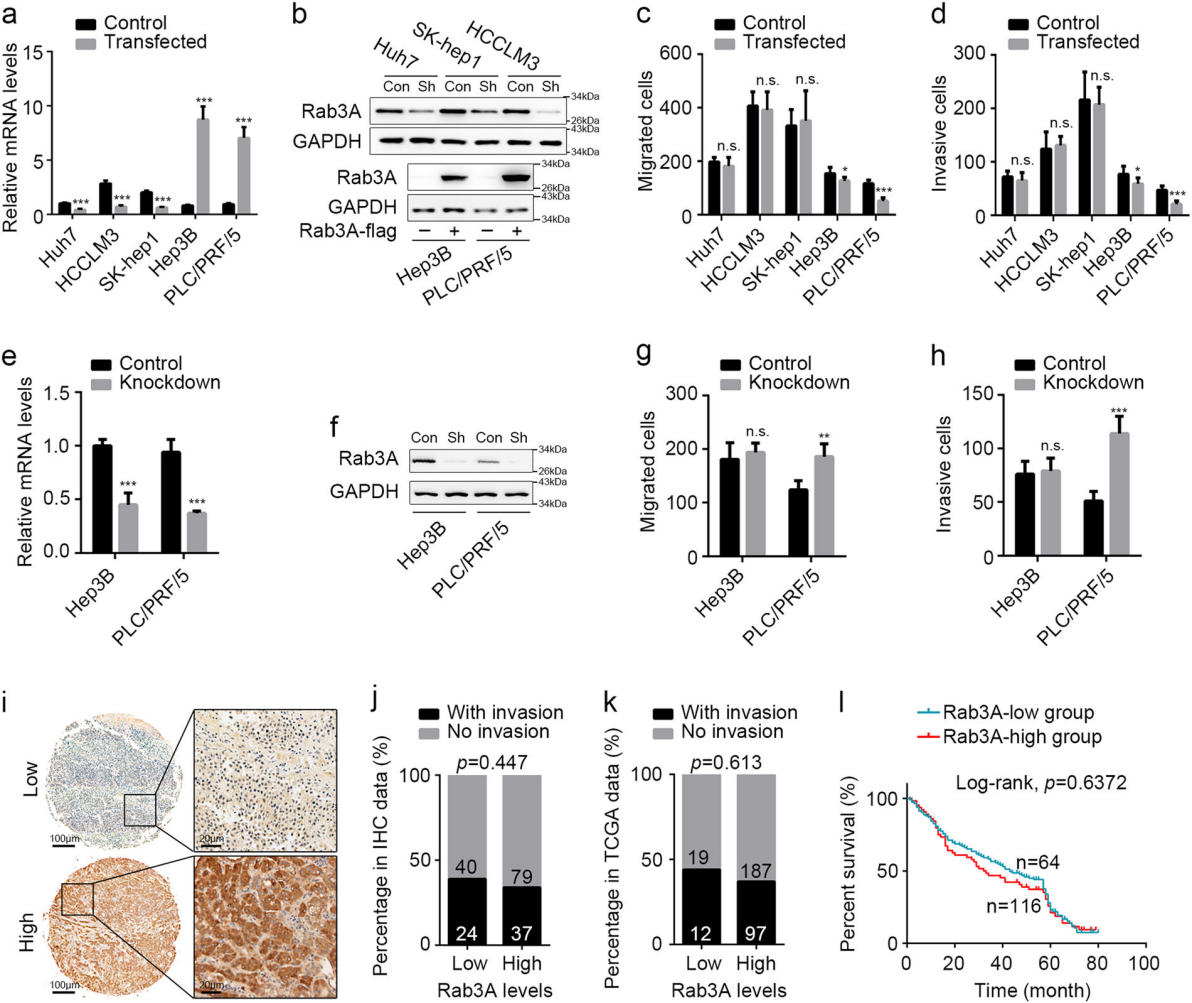
Upregulated Rab3A malfunctions in most HCC cells. **a**, **b** Efficiencies of Rab3A knockdown in Huh7 cells, SK-hep1 cells, and HCCLM3 cells, and Rab3A overexpression in Hep3B cells and PLC/PRF/5 cells were determined by qPCR and WB. GAPDH was used as internal control in both qPCR and WB analyses, and antibodies against Rab3A and GAPDH were used in WB. **c**, **d** Transwell assays for different HCC cells with Rab3A knockdown or overexpression. **e**, **f** Efficiencies of Rab3A knockdown in Hep3B cells and PLC/PRF/5 cells were determined by qPCR and WB. **g**, **h** Transwell assays for Hep3B and PLC/PRF/5 cells with Rab3A knockdown. **i** Representative IHC staining of tumor tissues with high or low levels of Rab3A in HCC. Regional magnification images were showed right. **j**, **k** Correlation between Rab3A expression and vessel invasion in IHC cohort and TCGA dataset. **l** Kaplan–Meier analysis for overall survival of HCC patients from IHC cohort. *p < 0.05, **p < 0.01, ***p < 0.001; NS, not significant

Considering the effects of Rab3A on metastasis in some other cancers^21–23^, we speculated that Rab3A might also regulate metastasis in HCC. As HCCLM3, SK-hep1, and Huh7 cells conferred relatively higher levels of Rab3A compared with Hep3B and PLC/PRF/5 cells (Additional file 1: Figure S1c-d), we knockdowned Rab3A in HCCLM3, SK-hep1, and Huh7 cells, and stably overexpressed Rab3A in Hep3B and PLC/PRF/5 cells (Fig. 2a–b and Additional file 2: Figure S2a). In vitro tests revealed that decreasing Rab3A in Huh7, SK-hep1, and HCCLM3 cells conferred no significant effects on migration and invasion, whereas overexpressing Rab3A in Hep3B and PLC/PRF/5 cells significantly inhibited migration and invasion (Fig. 2c–d), indicating that Rab3A is capable to inhibit HCC migration and invasion in Hep3B and PLC/PRF/5 cells, and there might be some regulatory factors suppressing the functions of Rab3A in other tumor cell lines. We further overexpressed Rab3A in Huh7 cells and decreased Rab3A in Hep3B and PLC/PRF/5 cells. Although Rab3A knockdown in PLC/PRF/5 cells was still able to promote migration and invasion, decreasing Rab3A in Hep3B cells did not influence the motility and invasion and overexpressing Rab3A in Huh7 cells led to significant changes on these effects (Fig. 2e–h and Additional file 2: Figure S2b–e). These results suggested that the regulatory factors targeting Rab3A might not be sufficient to inhibit the functions of Rab3A when the levels of Rab3A in HCC cells are too high to be manipulated, and PLC/PRF/5 cells might lack sufficient amount of these inhibitory factors targeting Rab3A. We also analyzed the clinical pathological characters of our IHC data and The Cancer Genome Atlas (TCGA) data after grouping the samples by Rab3A levels in ROC curve analysis (Fig. 2i). However, we failed to detect any significant correlations between vessel invasion and the expression of Rab3A in either mRNA levels or protein levels (Fig. 2j–k, and Additional file 3: Table S1). Furthermore, the upregulation of Rab3A was not associated with better overall survival of HCC patients (Fig. 2l). These data indicated that Rab3A is able to inhibit the migration and invasion of HCC cells, which is attenuated by some regulatory factors in most HCC cells, so that upregulated Rab3A malfunctions in regulating tumor invasion in most HCC tissues (Additional file 2: Figure S2f).

### *O*-GlcNAcylation suppresses the GTP-binding of Rab3A

**Fig. 3:**
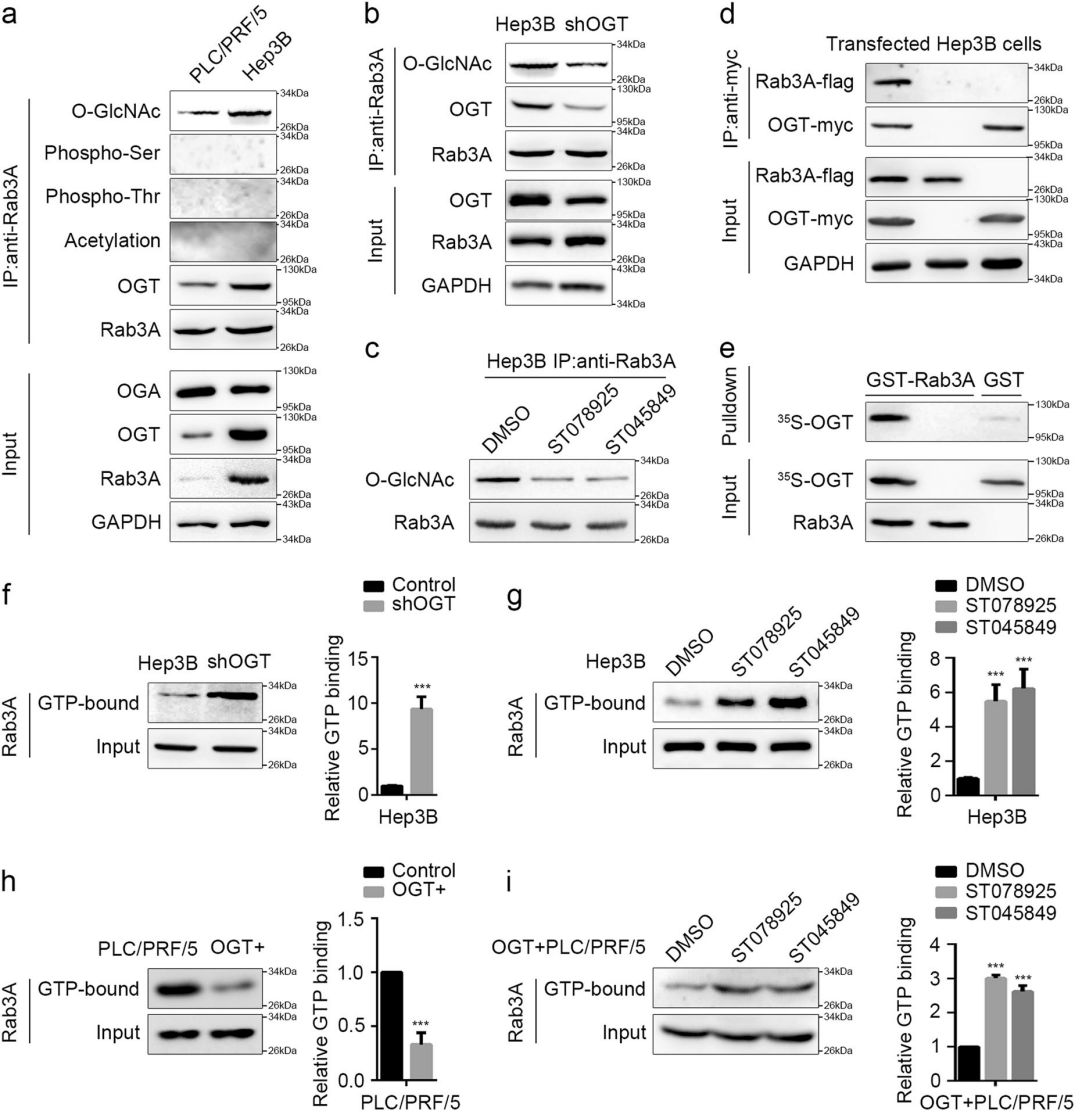
The modification of *O*-GlcNAcylation on Rab3A in HCC inhibits its GTP-binding affinity. **a** Modifications of Rab3A in PLC/PRF/5 cells and Hep3B cells were determined by IP analysis. **b** Interactions between Rab3A and OGT were determined by IP analysis in Hep3B cells with or without OGT knockdown. **c** *O*-GlcNAcylation levels of Rab3A were determined by IP analysis in Hep3B cells treated with DMSO, ST078925 (50 μM), or ST045849 (50 μM). **d** Interaction between transfected OGT-myc and Rab3A-flag in Hep3B cell was determined by IP analysis. **e** The interaction between Rab3A and OGT was determined by GST pull-down assay. **f** GTP pull-down assay for Hep3B cells with or without OGT knockdown. **g** GTP pull-down assay for Hep3B cells treated with DMSO, ST078925 (50 μM) or ST045849 (50 μM). **h** GTP pull-down assay for PLC/PRF/5 cells with or without OGT overexpression. **i** GTP pull-down assay for OGT-overexpressed PLC/PRF/5 cells treated with DMSO, ST078925 (50 μM), or ST045849 (50 μM). Representative Western blot analyses and statistical data were shown for all GTP pull-down assays (*n* = 6 independent experiments). ***p < 0.001

As posttranslational modifications are capable to modulate the functions of the RAS superfamily of GTPases including RABs^27^, we hypothesized that the inhibitory factors that attenuated Rab3A functions might be some modifications. The effectiveness of the antibody against phosphorylation or acetylation was confirmed in IP assays for transfected P53 in 293T cells (Additional file 4: Figure S3a). Our results revealed that *O*-GlcNAcylation instead of phosphorylation or acetylation on Rab3A was observed in both Hep3B and PLC/PRF/5 cells, and more *O*-GlcNAcylation on Rab3A was detected in Hep3B cells compared with that in PLC/PRF/5 cells (Fig. 3a). Considering the different effects of Rab3A in these two cell lines (Fig. 2g–h), the synchronous upregulation of Rab3A and its *O*-GlcNAcylation in Hep3B cells suggested that *O*-GlcNAcylation might be the key factor attenuating the effects Rab3A in suppressing migration and invasion. Our data also showed that OGT instead of OGA levels varied a lot between Hep3B and PLC/PRF/5 cells, and both OGT knockdown and OGT enzymatic-activity inhibition with ST078925^28^ or ST045849^28^ decreased the *O*-GlcNAcylation on Rab3A in Hep3B cells, indicating that aberrant *O*-GlcNAcylation of Rab3A in HCC cells mainly results from the changes of OGT levels (Fig. 3a–c and Additional file 4: Figure S3b–c). Furthermore, we observed the novel interaction between OGT and Rab3A in HCC cells, which was confirmed by GST pull-down assay (Fig. 3a–e and Additional file 4: Figure S3d). Further IP analysis on HCC tumor tissues and adjacent normal tissues revealed that Rab3A and *O*-GlcNAcylation were both increased in HCC tissues, and the *O*-GlcNAcylation levels of Rab3A was higher in tumor tissues than that in normal ones (Additional file 4: Figure S3e). Hyper-*O*-GlcNAcylation on Rab3A was also observed in HCC cell lines when compared with L02 cells (Additional file 4: Figure S3f).

*O*-GlcNAcylation could regulate phosphorylation, degradation, and GTP-binding properties of GTPases^8,29^. However, no phosphorylation was detected on Rab3A, and despite the synchronous level changes of Rab3A and its *O*-GlcNAcylation in different HCC cells, OGT knockdown failed to downregulate Rab3A levels (Fig. 3a–b), suggesting that *O*-GlcNAcylation might not regulate the potential signaling or the stability of Rab3A in HCC. Instead, OGT knockdown and OGT inhibitor treatment significantly increased Rab3A GTP-binding activity in Hep3B cells and OGT-overexpressed PLC/PRF/5 cells (*p* < 0.05) (Fig. 3f–i and Additional file 5: Figure S4a–c), indicating that elevated *O*-GlcNAcylation on Rab3A regulates the GTP-binding affinity of Rab3A in HCC cells.

### *O*-GlcNAcylation on Rab3A hinders the inhibition of Rab3A on HCC metastasis

**Fig. 4:**
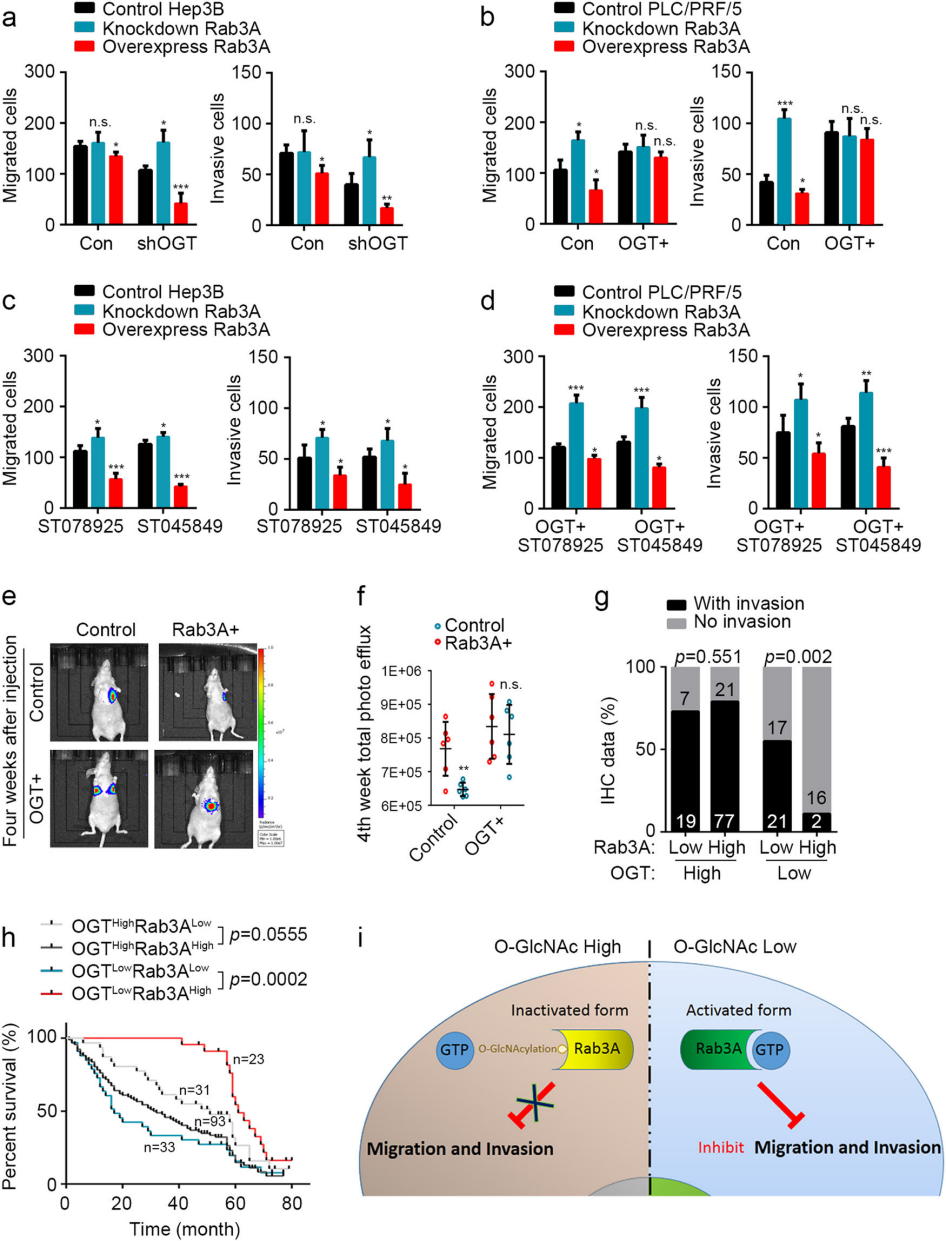
*O*-GlcNAcylation on Rab3A attenuates its inhibition on HCC metastasis. **a**, **b** Transwell assays for Hep3B and PLC/PRF/5 cells with Rab3A and/or OGT level changes. **c**, **d** Transwell assays for Hep3B-shOGT and PLC/PRF/5-OGT stable cells treated with ST078925 (50 μM) or ST045849 (50 μM). **e**, **f** In vivo effect of Rab3A in intravenous metastasis model at fourth week after injection (*n* = 6 in each group). Representative images of lung-metastasis mice were shown on the left, and statistical data was shown on the right. **g** Correlation of Rab3A and/or OGT expression with vessel invasion in IHC cohort. **h** Kaplan–Meier analysis for overall survival of HCC patients from IHC cohort. **i** A tentative model describing the potential suppressive effects of *O*-GlcNAcylation on Rab3A. *p < 0.05, **p < 0.01, ***p < 0.001; NS, not significant

To determine whether *O*-GlcNAcylation is the key regulator attenuating the effects of Rab3A on HCC metastasis, we knockdowned OGT in Hep3B cells and overexpressed OGT in PLC/PRF/5 cells before altering Rab3A levels in these cells (Fig. 4a–e and Additional file 5: Figure S4a–c). OGT knockdown in Hep3B cells revived the effects of Rab3A knockdown, and enhanced the inhibitory effects of overexpressed Rab3A on migration and invasion (Fig. 4a). In addition, in OGT-overexpressed PLC/PRF/5 cells, neither knockdown nor overexpression of Rab3A could regulate migration or invasion in vitro (Fig. 4b). Meanwhile, OGT inhibitors rescued the effects of Rab3A in Hep3B cells and OGT-overexpressed PLC/PRF/5 cells (Fig. 4c–d). In vivo data of intravenous inoculation also confirmed that overexpressing Rab3A in PLC/PRF/5 cells inhibited metastasis, whereas overexpressing OGT at the same time attenuated the effect of Rab3A (Fig. 4e–f). Further clinical analysis verified the significant difference in HCC metastasis between OGT^Low^Rab3A^High^ patients and OGT^Low^Rab3A^Low^ patients (*p* = 0.0017), and upregulated Rab3A in OGT^High^ HCC patients conferred no significant improvement in vessel invasion (*p* = 0.55138) (Fig. 4g, Additional file 6: Table S2 and Additional file 7: Figure S5). Survival analysis also showed that OGT^Low^Rab3A^High^ patients had better prognosis compared with OGT^Low^Rab3A^Low^ patients (Log-rank, *p* = 0.0002) and upregulated OGT dampened this improvement (Log-rank, *p* = 0.0555) (Fig. 4h). Taken together, these data demonstrated that *O*-GlcNAcylation hinders the Rab3A-mediated inhibition on HCC metastasis (Fig. 4i).

### OGT attenuates the effects of Rab3A on mitochondrial OXPHOS

**Fig. 5:**
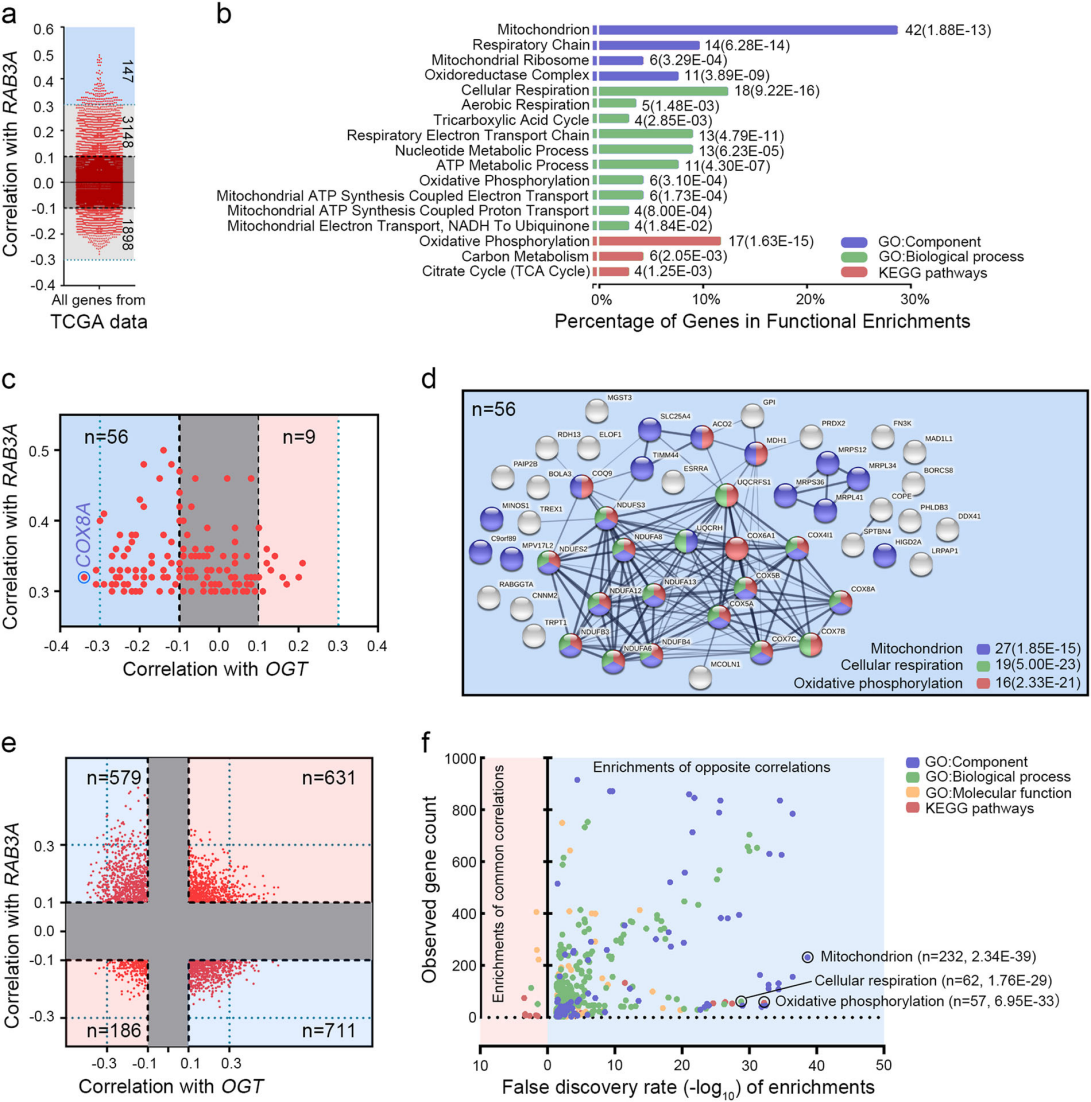
Genes correlated with *OGT* and *RAB3A* in HCC play opposite roles in mtOXPHOS. **a** The correlations of all genes from TCGA-LIHC dataset with *RAB3A*. **b** Functional enrichment for all the genes conferring moderate correlation with *RAB3A* (Pearson’s |R| > 0.3). **c** Correlation analysis between *OGT* and genes moderately correlated with *RAB3A*. **d** The co-expression network of 56 genes conferring positive correlation with *RAB3A* (Pearson’s *R* > 0.3) and negative correlation with *OGT* (Pearson’s *R* < − 0.1). **e** The correlations of all genes from TCGA-LIHC dataset with *OGT* and *RAB3A*. **f** Functional enrichment for the genes conferring opposite correlations with *OGT* and *RAB3A*

To investigate the mechanisms how Rab3A and its *O*-GlcNAcylation regulate HCC progression, we analyzed the co-expression genes with *RAB3A* and/or *OGT* in TCGA-LIHC dataset, and determined 147 genes moderately correlated with *RAB3A* (Pearson |R| > 0.3, *p* < 0.001) (Fig. 5a and Additional file 8: Table S3.1). Gene Ontology and Kyoto Encyclopedia of Genes and Genomes functional enrichments revealed that these genes were significantly associated with cellular respiration (18/147, *p* < 0.001), OXPHOS (17/147, *p* < 0.001), and mitochondrion (42/147, *p* < 0.001) (Fig. 5b, Additional file 8: Table S3.2 and Additional file 9: Figure S6a–d), indicating that *RAB3A* is involved in regulating mitochondrial OXPHOS (mtOXPHOS). Among these 147 genes, 56 of them were negatively correlated with *OGT* (Pearson’s |R| > 0.1, *p* < 0.001), more than OGT-positively correlated genes (*n* = 9), and mtOXPHOS-related pathways could still be enriched from these 56 genes (Fig. 5c–d, Additional file 8: Table S3.3 and Additional file 9: Figure S6e). Further analysis on all genes correlated with both *OGT* and *RAB3A* (|R| > 0.1, *p* < 0.001, *n* = 2107) also revealed that most of them (1290/2107) conferred opposite correlations with Rab3A and OGT, and these oppositely correlated genes were associated with 456 functionally-enriched pathways including mitochondrion, cellular respiration, and OXPHOS, whereas only 11 pathways could be enriched from the rest genes (817/2107) (Fig. 5e–f and Additional file 8: Table S3). These data suggested that Rab3A and its *O*-GlcNAcylation might have opposite roles in different biological pathways, particularly mtOXPHOS.

**Fig. 6:**
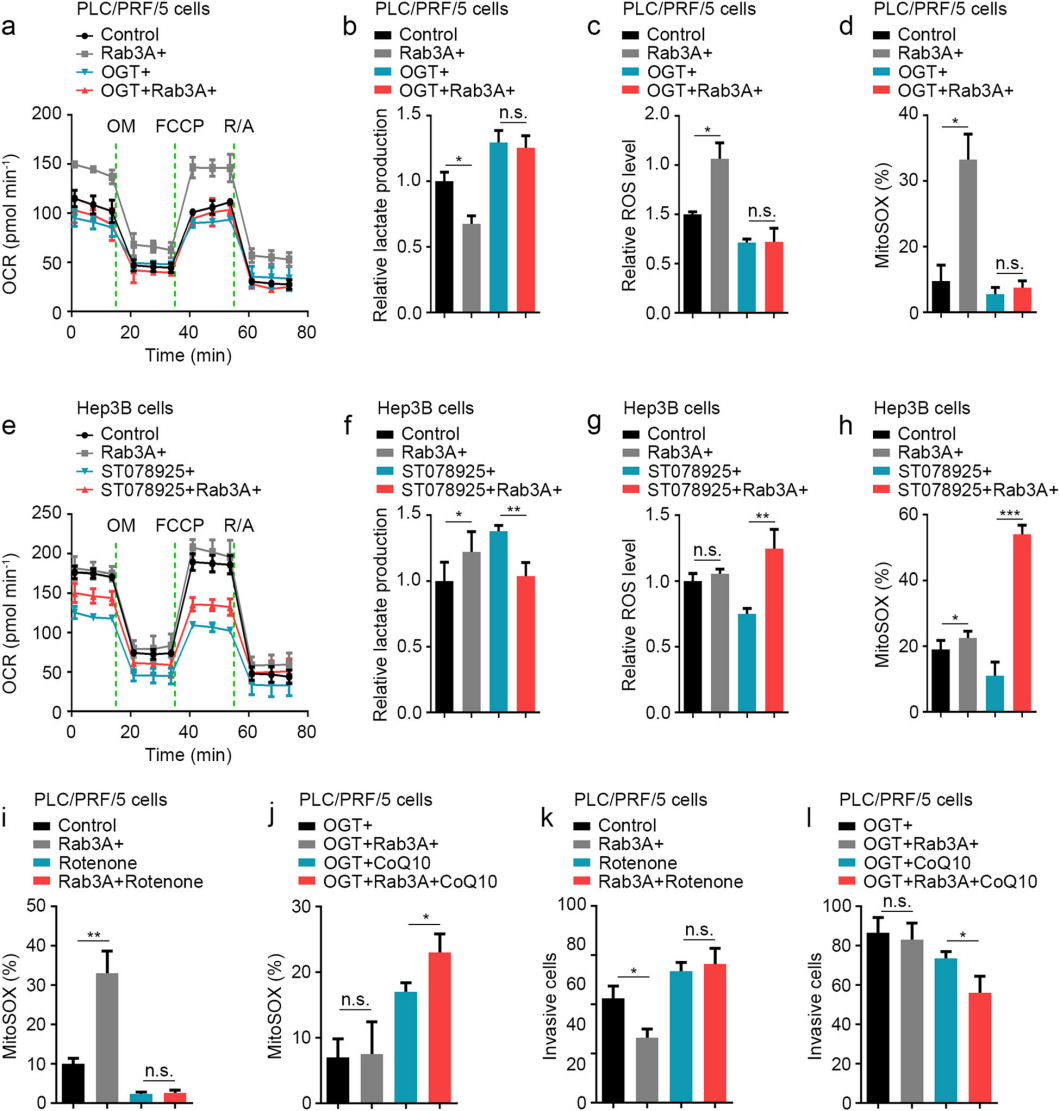
*O*-GlcNAcylation attenuates the effect of Rab3A on metastasis via hindering mtOXPHOS. **a** The OCR curves in control, Rab3A + , OGT + , and OGT + Rab3A + PLC/PRF/5 cells. **b** Lactate production normalized to the cell numbers in control, Rab3A + , OGT + , and OGT + Rab3A + PLC/PRF/5 cells. **c** ROS levels were determined by ROS-Glo assays in control, Rab3A + , OGT + , and OGT + Rab3A + PLC/PRF/5 cells. **d** MitoSOX Red staining of control, Rab3A + , OGT + , and OGT + Rab3A + PLC/PRF/5 cells were analyzed by flow cytometry. **e** OCR curves in control and Rab3A + Hep3B cells treated with DMSO or ST078925 (50 μM). **f** Lactate production normalized to the cell numbers in control and Rab3A + Hep3B cells treated with DMSO or ST078925 (50 μM). **g** ROS levels were determined by ROS-Glo assays in control and Rab3A + Hep3B cells treated with DMSO or ST078925 (50 μM). **h** MitoSOX Red staining of control and Rab3A + Hep3B cells treated with DMSO or ST078925 (50 μM) were analyzed by flow cytometry. **i**, **j** MitoSOX Red staining of PLC/PRF/5 cells with indicated treatments. **k**, **l** Transwell invasion assays for PLC/PRF/5 cells with indicated treatments. Rotenone (0.5 μM) or CoQ10 (10 μM) was used in some assays. Six independent experiments were performed for each assay. *p < 0.05, **p < 0.01, ***p < 0.001; NS, not significant

To verify the effects of Rab3A and OGT on mtOXPHOS, we examined the mitochondrial respiratory capacity in vitro. In PLC/PRF/5 cells, Rab3A elevated the basal and maximal respiratory capacities, whereas OGT attenuated the effect of Rab3A (Fig. 6a). Furthermore, the decrease of lactate production resulting from Rab3A was also weakened by OGT (Fig. 6b). Meanwhile, as reactive oxygen species (ROS) can be elevated through activation of the electron transport chain in mitochondria, we measured levels of ROS as well, and we found that H_2_O_2_ and mitochondrial superoxide levels were elevated by Rab3A, in line with a hyperactive mtOXPHOS, which was dampened by OGT (Fig. 6c–d). Similar with these results in PLC/PRF/5 cells, ST078925 enhanced the effects of Rab3A on OCR and ROS in Hep3B cells (Fig. 6e–h). Meanwhile, overexpressing Rab3A in untreated Hep3B cells promoted lactate production, whereas ST078925 treatment reversed this effect and mediated the suppressive function of Rab3A on lactate production (Fig. 6f). These data confirmed that Rab3A-enhanced mtOXPHOS is hindered by high levels of *O*-GlcNAcylation in most HCC cells.

### Rab3A and its *O*-GlcNAcylation regulate HCC metastasis via modulating mtOXPHOS

Despite the well-known relations between respiration and metastasis, it is still unclear whether mitochondrial respiration drives metastasis, or vice versa. To test this cause-effect relationship, we used rotenone^30^, a specific inhibitor of the mitochondrial electron transport complex I, to block mtOXPHOS in PLC/PRF/5 cells. We also used coenzyme Q10 (CoQ10)^31^, an agonist enhancing electron transporting, to elevate mtOXPHOS in PLC/PRF/5 cells. Mitochondrial superoxide levels were assessed to determine the effects of rotenone and CoQ10 on mtOXPHOS, and the data showed that rotenone totally abolished the elevation of Rab3A on MitoSOX levels, whereas CoQ10 promoted the overall mtOXPHOS (Fig. 6i–j). Moreover, rotenone-induced respiration inhibition attenuated the functions of Rab3A on invasion, and CoQ10 treatment partially rescued the impaired effects of Rab3A resulted from its *O*-GlcNAcylation (Fig. 6k–l). These observations suggested that Rab3A and its *O*-GlcNAcylation might regulate HCC metastasis via modulating mtOXPHOS.

### COXs are critical for the functions of Rab3A and its *O*-GlcNAcylation in HCC

**Fig. 7:**
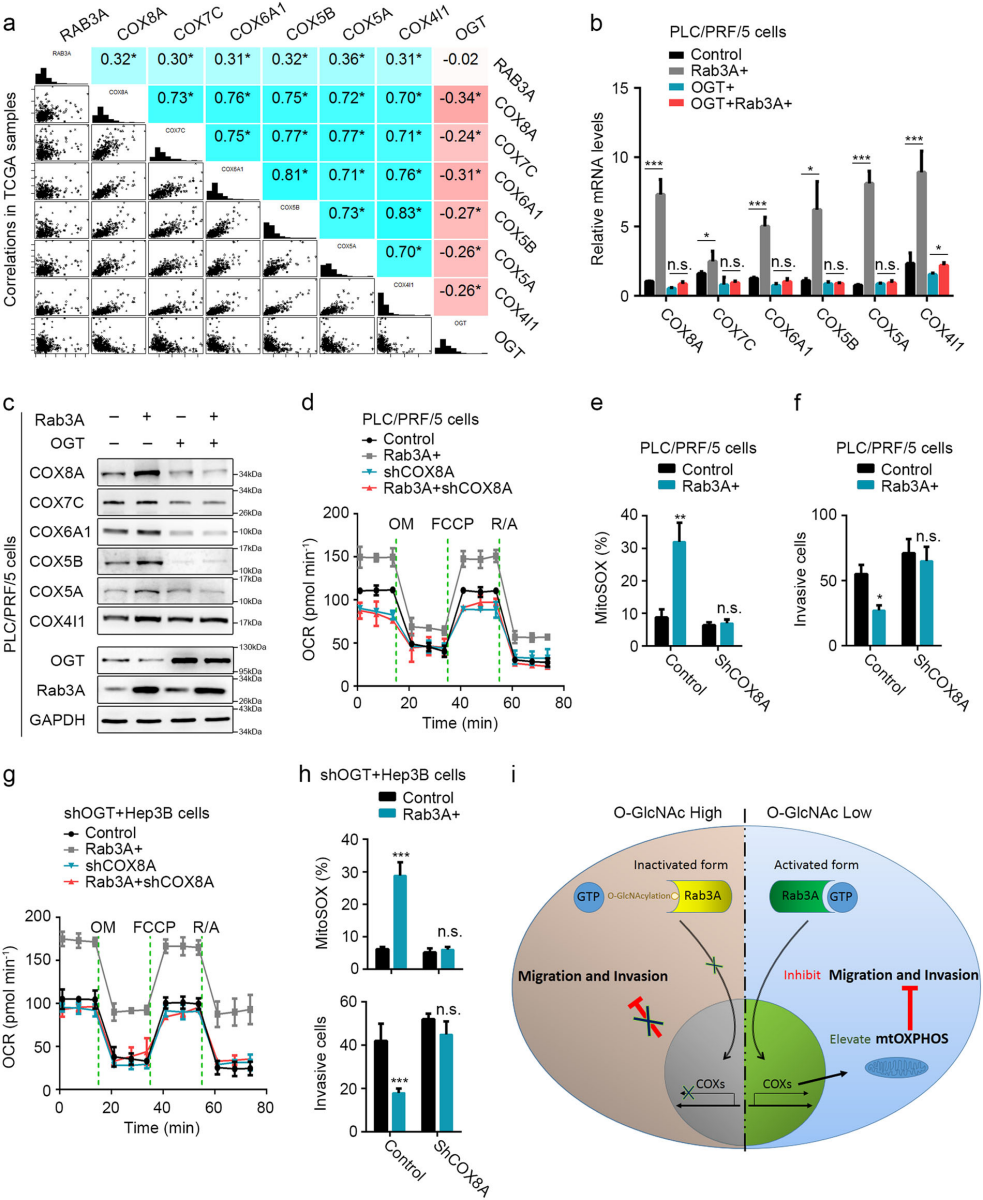
COXs are critical for the functions of Rab3A and its *O*-GlcNAcylation in HCC. **a** mRNA correlations of Rab3A, OGT, COX8A, COX7C, COX6A1, COX5B, COX8A, and COX4I1 in 442 primary HCC tumors of TCGA (*n* = 442 biologically independent patient samples). **b**, **c** mRNA and protein levels of COX8A, COX7C, COX6A1, COX5B, COX5A, and COX4I1 in PLC/PRF/5 cells with OGT and/or Rab3A expression. **d** The OCR curves in parental PLC/PRF/5, Rab3A + , shCOX8A, and Rab3A + shCOX8A cells treated with oligomycin, FCCP, and rotenone/antimycin A. **e** MitoSOX Red staining of PLC/PRF/5 cells with indicated treatments was analyzed by flow cytometry. **f** Transwell invasion assay for PLC/PRF/5 cells with indicated treatments. Six independent experiments were performed for each assay. **g** The OCR curves in OGT-knockdowned Hep3B, shOGT + Rab3A + , shOGT + shCOX8A, and shOGT + Rab3A + shCOX8A cells treated with oligomycin, FCCP, and rotenone/antimycin A. **h** MitoSOX Red staining of HeprB cells with indicated treatments was analyzed by flow cytometry, and the invasive ability was analyzed by transwell invasion assays under indicated treatments. **i** The schematic diagram describing the function of Rab3A and *O*-GlcNAcylation in HCC metastasis. Six independent experiments were performed for each assay. *p < 0.05, **p < 0.01, ***p < 0.001; NS, not significant

COX is the multimeric enzyme that executes the last step in mtOXPHOS and dysregulation of COX induces Warburg effect and metabolic reprogramming in cancers^32^. Moreover, COX reduction was observed during epithelial-to-mesenchymal transition^33^ and COX knockdown could transform malignant cells into invasive phenotypes^32^. In HCC, several COX subunits (COXs) were significantly correlated with Rab3A and OGT (Fig. 7a). In addition, Rab3A elevated the mRNA levels of these COXs in PLC/PRF/5 cells, which was attenuated by overexpressed OGT (Fig. 7b). Similar WB results verified this observation in protein levels (Fig. 7c). Knocking down COX8A, one of these Rab3A-correlated COXs, in PLC/PRF/5 cells, eliminated the effects of Rab3A on basal respiratory capacities and mitochondrial superoxide levels (Fig. 7d–e and additional file 10: Figure S7a–e). Furthermore, COX8A knockdown offset the inhibition of Rab3A on invasion (Fig. 7f). Similar results were observed in OGT-KD Hep3B cells (Fig. 7g–h and Additional file 10: Figure S7c-g), confirming that COX8A could be regulated by Rab3A and OGT, and COX8A knockdown could dispel the effects of Rab3A on MitoSOX and invasion in OGT-KD Hep3B cells. These data indicated that COXs are crucial for the functions of Rab3A in both mtOXPHOS and metastasis (Fig. 7i).

## Discussion

Tumor metastasis is widely observed in human malignancies^34^. During metastasis, imbalances in dynamic vesicle trafficking processes are critical for the initiation of tumor cell transformation and invasion^13^. In addition, deregulated trafficking pathways in cancers usually result from the aberrant expressions or functions of Rab proteins that are essential components of vesicle trafficking machinery^15^. In this study, we observed the upregulation of Rab3A in HCC. However, different from its carcinogenic roles in nervous system neoplasms^35^ and breast cancer^20,36^, our data indicated that Rab3A functions as a tumor suppressor in HCC. Some other Rabs such as Rab25 also confers similar contrary effects in different cancers^37–39^. This phenomenon might result from Rab-associated cell-type-specific distinct downstream pathways or regulators in different tissues. Our data here revealed that for Rab3A in HCC, the key downstream pathway is mtOXPHOS and the critical regulatory effector is *O*-GlcNAcylation.

Emerging evidences suggest that hyper-*O*-GlcNAcylation in cancers is involved in metastasis. In HCC, hyper-*O*-GlcNAcylation is associated with tumor recurrence and increasing *O*-GlcNAcylation in HCC cells enhances migration and invasion^29,40,41^. Although hyper-*O*-GlcNAcylation depends on both OGT and OGA^42^, our data suggested that aberrant expression of OGT instead of OGA might be responsible for hyper-*O*-GlcNAcylation in HCC. In addition, decreasing OGT levels or using OGT inhibitor could reduce the overall *O*-GlcNAc levels in HCC, which finally influenced the migration/invasion of HCC cells^40,43^.

Although *O*-GlcNAcylation could regulate the stabilization or degradation of metastasis-associated proteins^29^ and modulate metastasis-associated signaling via interplay with phosphorylation^8^, we failed to detect influenced stability or altered phosphorylation of Rab3A by *O*- GlcNAcylation. Instead, *O*-GlcNAcylation decreases the GTP-binding affinity of Rab3A. This effect of *O*-GlcNAcylation has been reported on some other GTPases^44,45^. Similar with their reports, our results also suggest that *O*-GlcNAcylation decreases GTP-binding affinity independent of phosphorylation. And due to the indispensable requirement of the GTP-binding property of Rabs for its biological functions^14^, this effect of *O*-GlcNAcylation could attenuate the inhibition of Rab3A on metastasis, and Rab3A could only function when its protein levels are beyond the saturation of *O*-GlcNAcylation. Therefore, hyper-*O*-GlcNAcylation in most HCC tumor cells increases the threshold level, and the effects of endogenous Rab3A in HCC cell lines are dampened as revealed in our study. Moreover, due to the ubiquitous hyper-*O*-GlcNAcylation in HCC patients, Rab3A malfunctions in most HCC patients despite its upregulation.

In addition to the role in metastasis, we verified the effects of Rab3A and its *O*-GlcNAcylation on metabolic reprogramming. Rab3A promotes the expression of se`veral COXs to enhance mtOXPHOS and reverse Warburg Effect in HCC, whereas hyper-*O*-GlcNAcylation inhibits this effect of Rab3A. *O*-GlcNAcylation regulates metabolic reprograming in different mechanisms, such as directly regulating metabolic pathways^46^ or modulating metabolism-related signal pathways^43^, which finally facilitates the metastasis of tumor cells^9^. Rabs were also reported to function in regulating metabolism. Some Rabs regulate the GLUT4 translocation to enhance glucose uptake, and some Rabs could modulate the target of rapamycin complex signaling to regulate metabolism indirectly^47^. Although the function of Rab3A in metabolism has never been reported, some evidences suggested the potential role of Rab3A in regulating metabolism, especially mitochondrion oxidative metabolism. Reduced Rab3A protein levels were observed synchronously with decreased ATP levels and ROS production in rosuvastatin-treated neurons^48^. In addition, the proteomics analysis on pancreatic β-cells from type 2 diabetes patients also showed similar synchronous upregulation of Rab3A with some mitochondrial metabolism-related proteins including several COXs^49^. Moreover, silencing Rab3A could block the effects of GLP-1 on enhancing cellular glucose uptake, mitochondrial membrane potential, and cellular ATP levels in β-cells^50^. Combined with our results, these data suggest the universal promotion of Rab3A on cellular respiration and mtOXPHOS in different cells. Furthermore, although we failed to detect the phosphorylation of Rab3A here, its universal regulation on the transcription of different COXs suggests that the function of Rab3A in metabolism reprogramming might be also mediated by its signaling, which requires further investigation.

Our data also suggest that Rab3A inhibits metastasis via enhancing mtOXPHOS. Mitochondrial oxidative metabolism has been reported to suppress metastasis in different cancers including HCC^51^. On one hand, ROS generated by OXPHOS limits metastasis directly^52^. On the other hand, mitochondrial metabolism is associated with the activity of p53^53^ and PTEN^54^, which further inhibits metastasis. Our data here provide extra evidences for the harmful role of elevated mtOXPHOS in HCC metastasis, and suggests that Rab3A is critical in this process. Intriguingly, the opposite roles of Rab3A in different cancers and its universal effects on OXPHOS suggest that mitochondrial oxidative metabolism might also play opposite roles in different cancers. Additional studies will be required to elucidate the detailed mechanisms underling these phenomena.

In summary, we found that despite the upregulation in HCC, Rab3A functions as a tumor suppressor in HCC metastasis and metabolic reprogramming. Rab3A elevates the expression of some COXs to promote mtOXPHOS, which consequently attenuates HCC metastasis. Hyper-*O*-GlcNAcylation on Rab3A in most HCC cells attenuates these suppressive effects of Rab3A via decreasing the GTP-binding affinity of Rab3A. Further studies on downstream signaling of Rab3A and the effects of *O*-GlcNAcylation on Rab3A in other cancers are required.

## Electronic supplementary material

Figure S1

Figure S2

Table S1

Figure S3

Figure S4

Table S2

Figure S5

Table S3

Figure S6

Figure S7

Supplementary figure legends
