# Supplementary figures and images for: O-GlcNAcylation on Rab3A attenuates its effects on mitochondrial oxidative phosphorylation and metastasis in hepatocellular carcinoma
Source: Cell Death Dis. 2018 Sep 20;9(10):970. doi: 10.1038/s41419-018-0961-7 (PMC6148238; doi:10.1038/s41419-018-0961-7)

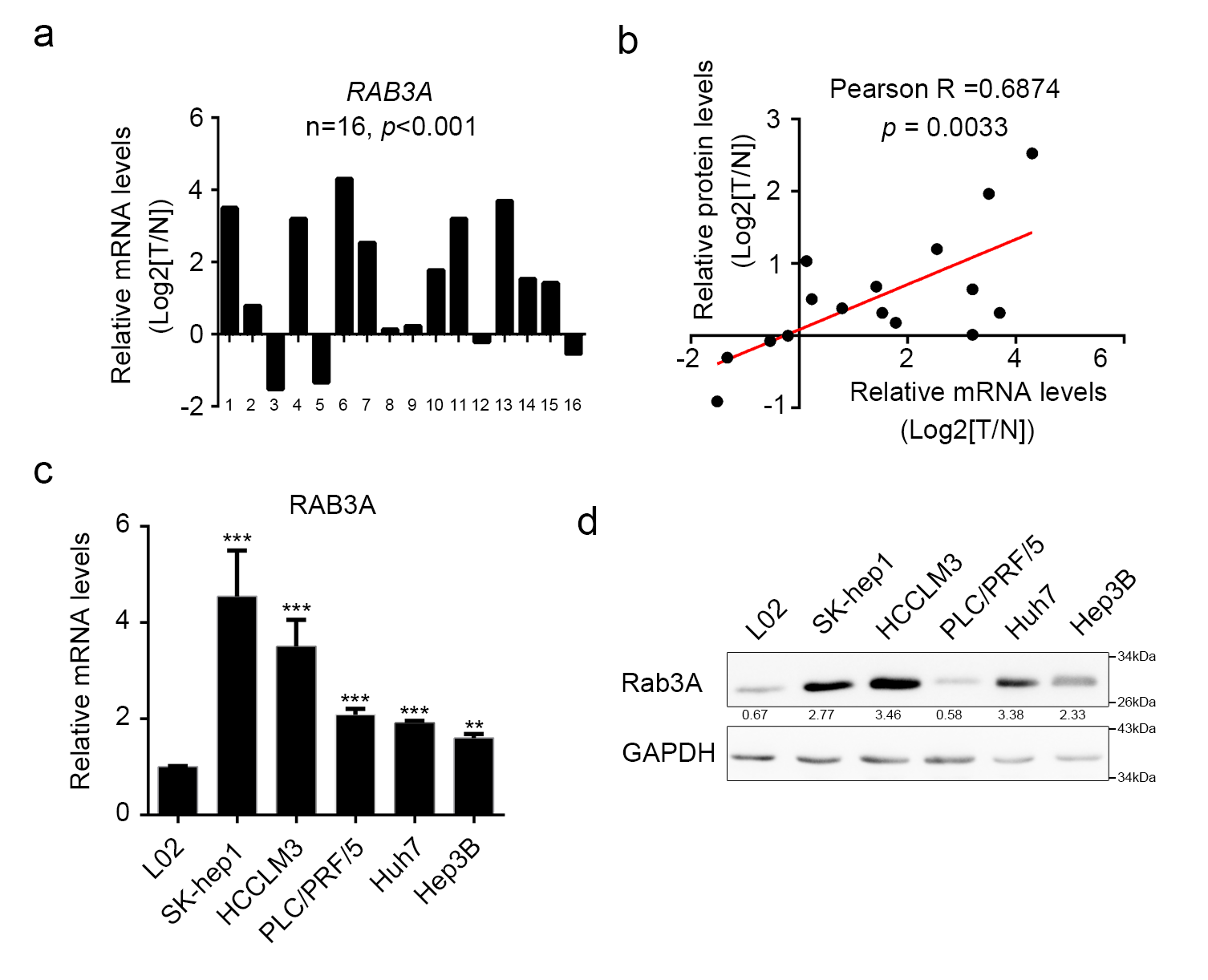

Supplement: Supplementary file 1 — Figure S1 [file 41419_2018_961_MOESM1_ESM.tif]

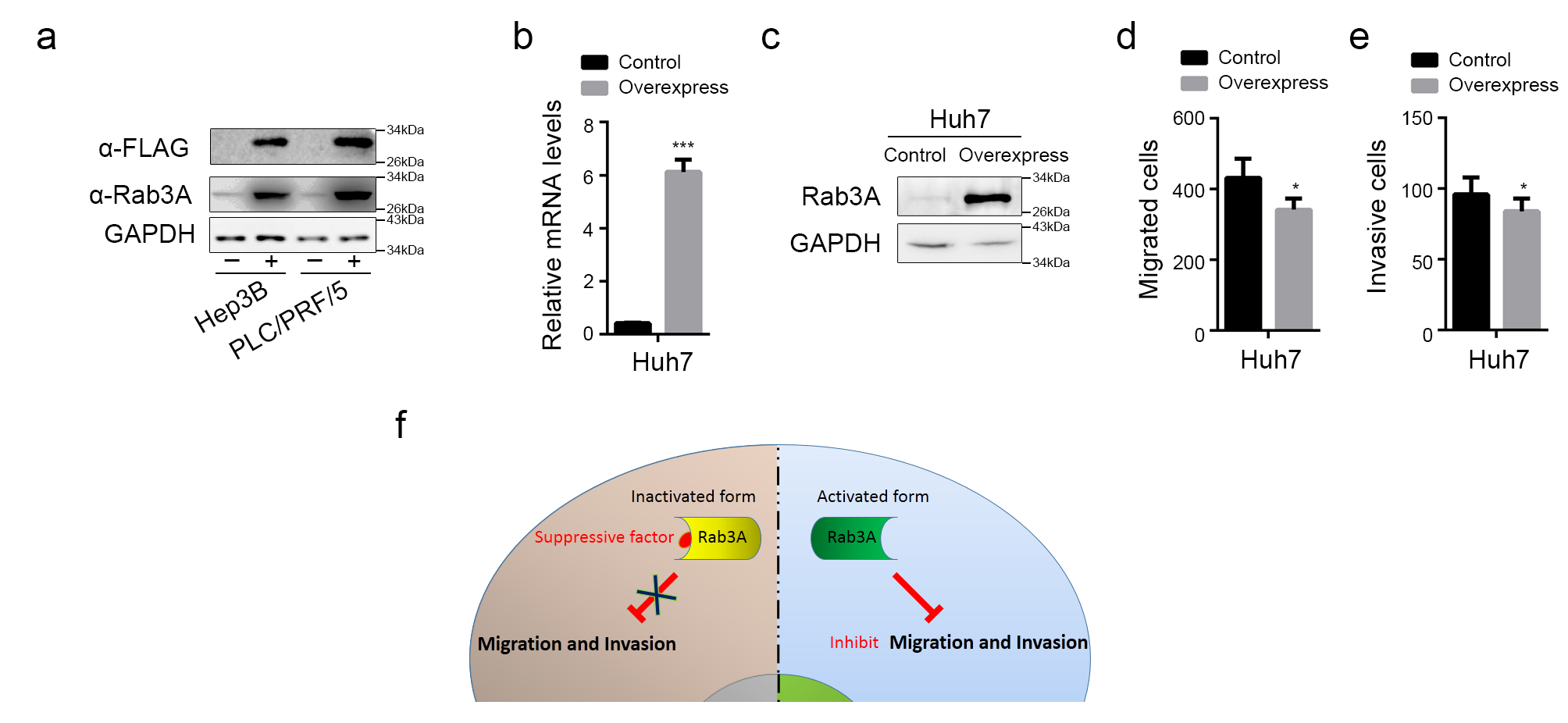

Supplement: Supplementary file 2 — Figure S2 [file 41419_2018_961_MOESM2_ESM.tif]

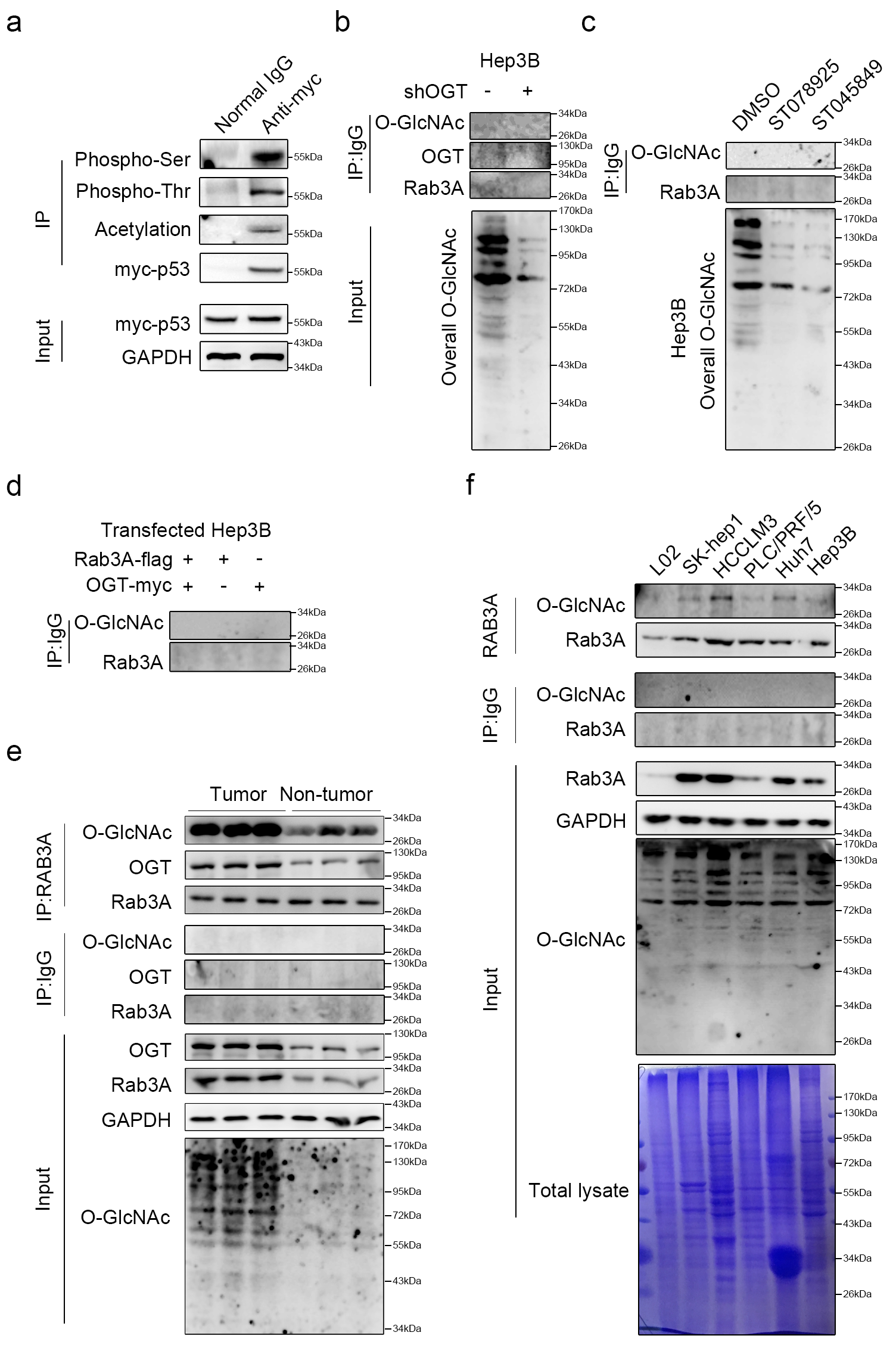

Supplement: Supplementary file 4 — Figure S3 [file 41419_2018_961_MOESM4_ESM.tif]

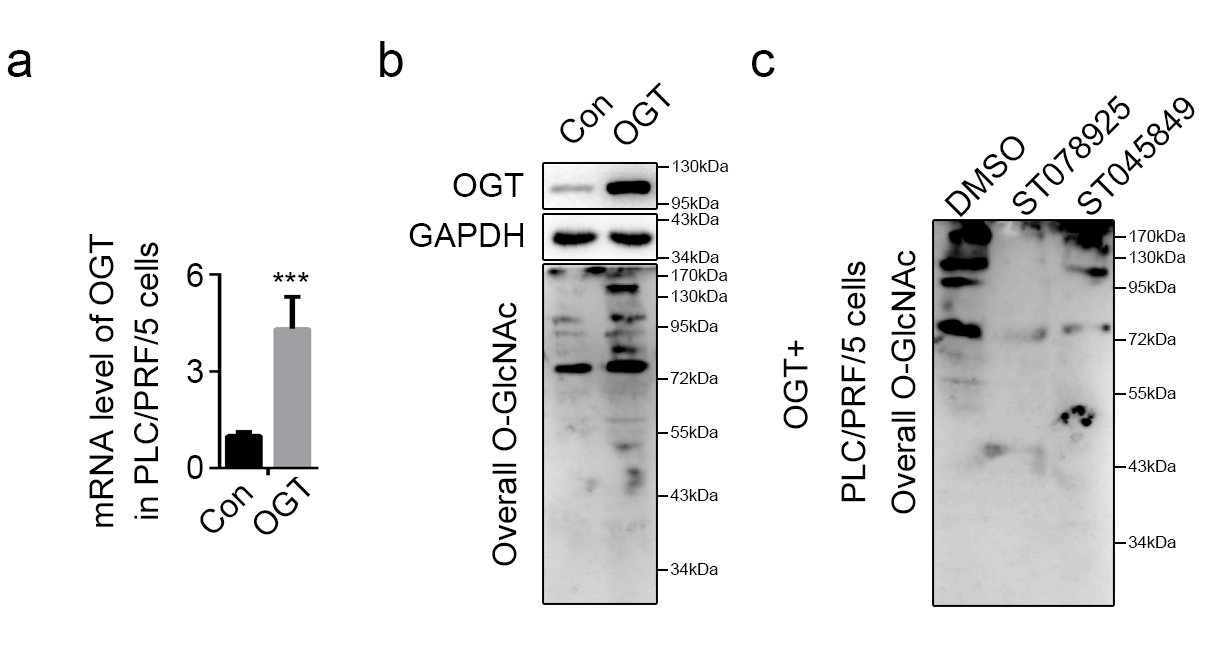

Supplement: Supplementary file 5 — Figure S4 [file 41419_2018_961_MOESM5_ESM.tif]

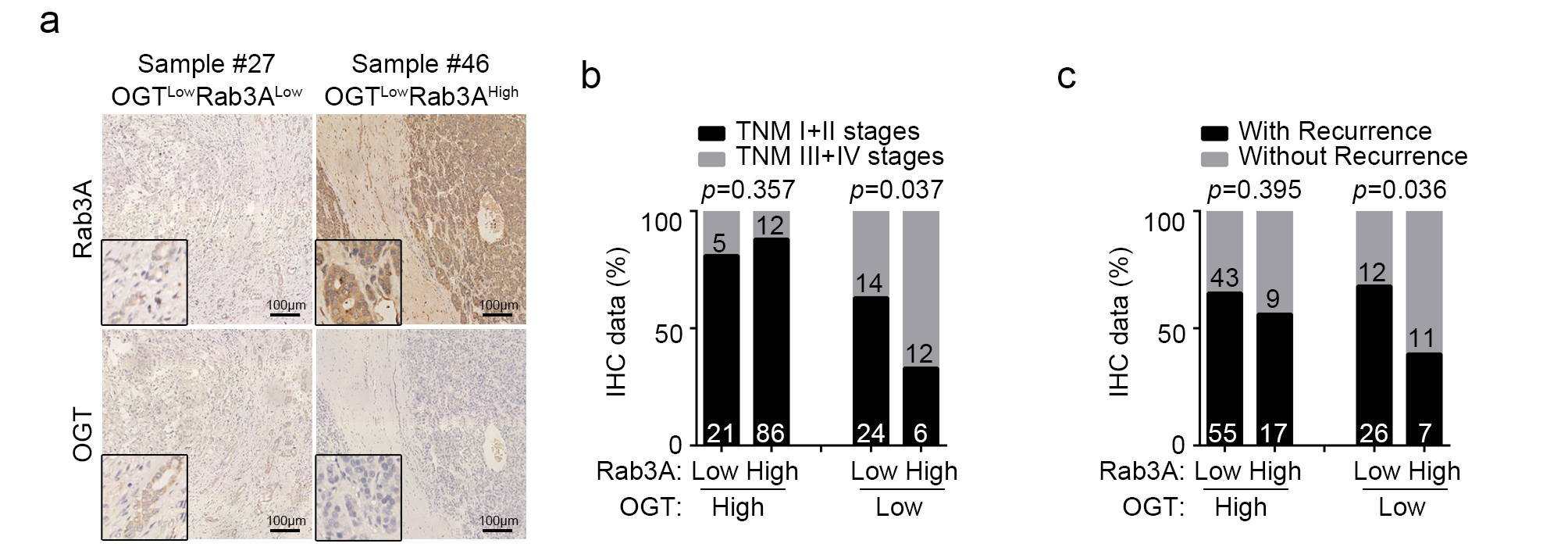

Supplement: Supplementary file 7 — Figure S5 [file 41419_2018_961_MOESM7_ESM.tif]

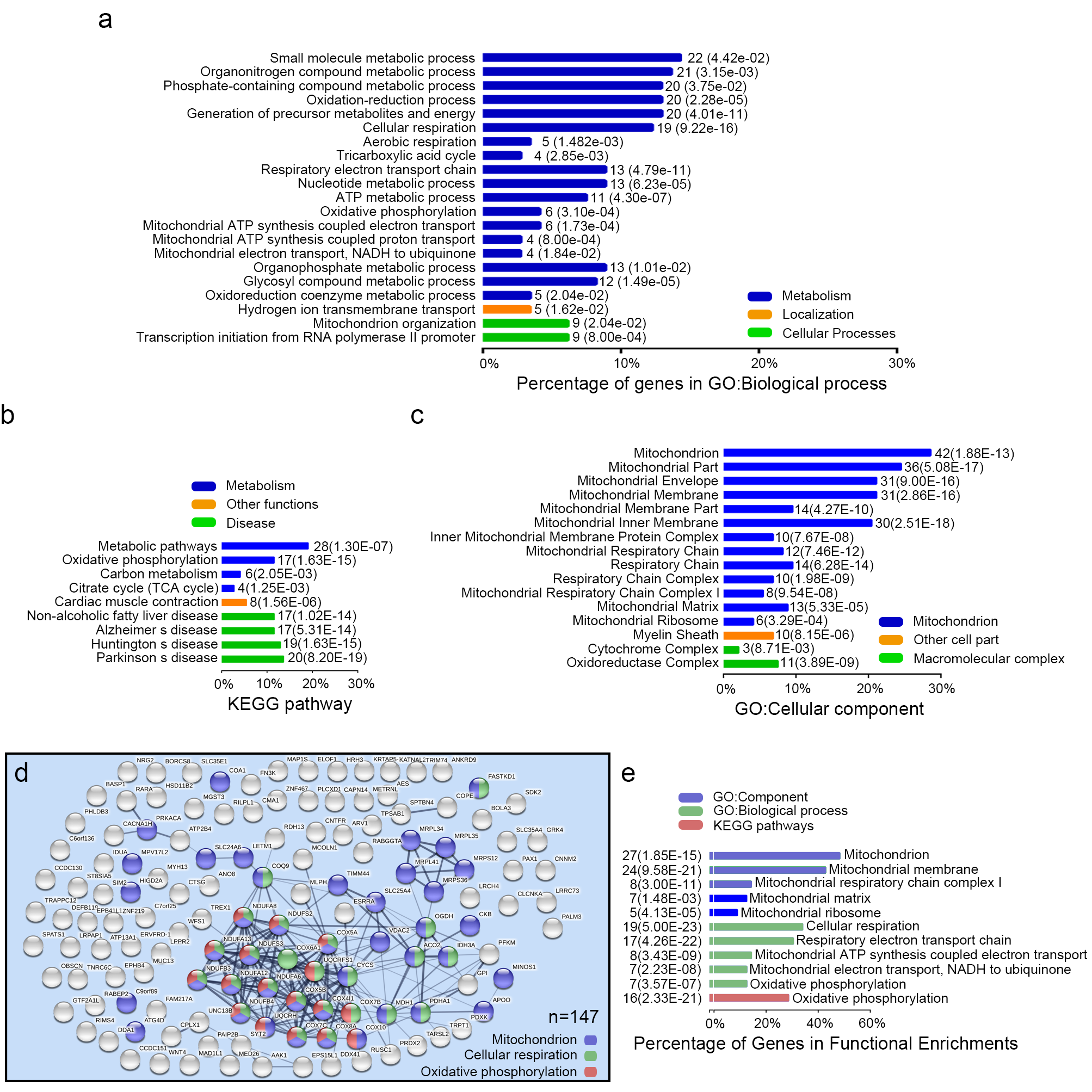

Supplement: Supplementary file 9 — Figure S6 [file 41419_2018_961_MOESM9_ESM.tif]

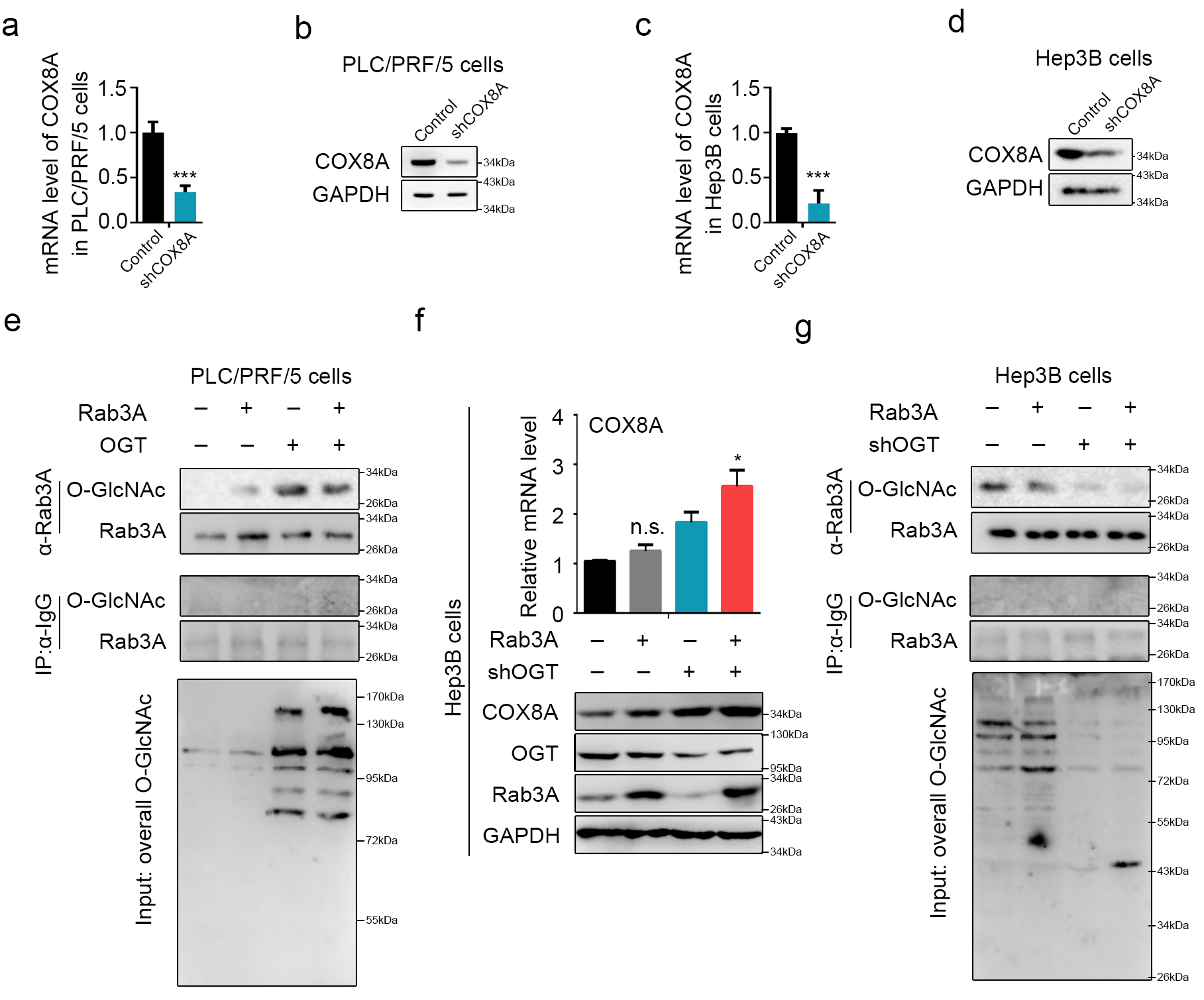

Supplement: Supplementary file 10 — Figure S7 [file 41419_2018_961_MOESM10_ESM.tif]
