# Supplementary material for: O-GlcNAcylation on Rab3A attenuates its effects on mitochondrial oxidative phosphorylation and metastasis in hepatocellular carcinoma
Source: Cell Death Dis. 2018 Sep 20;9(10):970. doi: 10.1038/s41419-018-0961-7 (PMC6148238; doi:10.1038/s41419-018-0961-7)
Supplement: Supplementary file 3 — Table S1 [file 41419_2018_961_MOESM3_ESM.docx]

**Table S1. Relationships between the Rab3A expression and the clinicopathological variables of hepatocellular carcinoma patients**

|  |  | Rab3A Expression | |  |
| --- | --- | --- | --- | --- |
|  |  | high | low |  |
| **Variables** | **No.** | **No. (%)** | **No. (%)** | ***P*-value** |
| **Gender** |  |  |  | 0.4471 |
| Male | 146 | 96(66%) | 50(34%) |  |
| Female | 34 | 20(59%) | 14(41%) |  |
| **Age (year)** | |  |  | 0.56015 |
| >52 | 96 | 60(63%) | 36(38%) |  |
| <=52 | 84 | 56(67%) | 28(33%) |  |
| **Vessel invasion** | |  |  | 0.44708 |
| Present | 119 | 79(66%) | 40(34%) |  |
| Absent | 61 | 37(61%) | 24(39%) |  |
| **Tumor size (cm)** | |  |  | 0.18700 |
| >5 | 121 | 74(61%) | 47(39%) |  |
| <=5 | 59 | 42(71%) | 17(29%) |  |
| **TNM stage** |  |  |  | 0.17535 |
| I+II | 137 | 92(67%) | 45(33%) |  |
| III+IV | 43 | 24(56%) | 19(44%) |  |
| **Recurrence** | |  |  | 0.07349 |
| Present | 105 | 62(59%) | 43(41%) |  |
| Absent | 75 | 54(72%) | 21(28%) |  |
| **Liver cirrhosis** | |  |  | 0.70523 |
| Present | 112 | 71(63%) | 41(37%) |  |
| Absent | 68 | 45(66%) | 23(34%) |  |
|  |  |  |  |  |

*p*<0.05 indicates that differences have statistical significance. *Pearson chi-square tests.
