# Supplementary material for: O-GlcNAcylation on Rab3A attenuates its effects on mitochondrial oxidative phosphorylation and metastasis in hepatocellular carcinoma
Source: Cell Death Dis. 2018 Sep 20;9(10):970. doi: 10.1038/s41419-018-0961-7 (PMC6148238; doi:10.1038/s41419-018-0961-7)
Supplement: Supplementary file 11 — Supplementary figure legends [file 41419_2018_961_MOESM11_ESM.docx]

**Supplementary figure legends**

**Fig. S1 Rab3A levels in different HCC cell lines**

**a** Relative mRNA level changes of Rab3A in 16 pairs of HCC tumor tissues and adjacent non-tumor tissues was determined by real-time PCR. **b** The correlation between mRNA level changes and protein level changes of 16 pairs of HCC tumor tissues and adjacent non-tumor tissues. **c,** **d** The mRNA (c) and protein levels (d) of Rab3A in L02 and five HCC cell lines were determined by qPCR and Western blot.

**Fig. S2 Overexpressing Rab3A in Huh7 cells still inhibits migration and invasion.**

**a-c** Overexpression efficiency of Rab3A in Hep3B, PLC/PRF/5, and Huh7 cells was determined by qPCR and Western blot. **c, d** The effects of overexpressed Rab3A on migration and invasion of Huh7 cells were determined by Transwell assays. **f** A tentative model describing different functions of Rab3A in different HCC cells.

**Fig. S3 Effectiveness of the antibodies in IP assays**

**a** Effectiveness of the antibody against phosphorylation or acetylation was confirmed in IP assays for transfected P53 in 293T cells. **b-d** Normal IgG was used as control in different IP analysis. And input *O*-GlcNAcylation levels were determined at the same time. **e** IP analysis on the *O*-GlcNAcylation of Rab3A in 3 pairs of HCC tumor tissues and adjacent non-tumor tissues. **f** IP analysis on the *O*-GlcNAcylation of Rab3A in different HCC cell lines.

**Fig. S4 OGT in PLC/PRF/5 cells increases overall *O*-GlcNAcylation levels.**

**a, b** Efficiency of OGT stable overexpression in PLC/PRF/5 cells was determined by qPCR and Western blot. **c** ST078925 (50 μM) and ST045849 (50 μM) reduced overall *O*-GlcNAcylation levels in OGT-overexpressed PLC/PRF/5 cells.

**Fig. S5 Upregulated Rab3A is associated with higher TNM stage and more recurrences in OGT^low^ patients.**

**a** Reprehensive staining of OGT and Rab3A in OGT^Low^Rab3A^Low^ and OGT^Low^Rab3A^High^ patients. **b, c** Correlations of Rab3A expression with TNM stage and HCC recurrence in IHC cohort.

**Fig. S6 Functional enrichments for Rab3A and/or OGT.**

**a-c** Functional enrichments of GO and KEGG pathway for 147 Genes moderately correlated with Rab3A (Pearson R>0.3). **d** The co-expression network of 147 genes conferring positive correlation with RAB3A (Pearson R>0.3). **e** Functional enrichments of GO and KEGG pathway for 56 genes conferring positive correlation with *RAB3A* (Pearson R>0.3) and negative correlation with *OGT* (Pearson R<-0.1).

**Fig. S7 Knockdown efficiency of COX8A in PLC/PRF/5 cells.**

**a-d** Knockdown efficiency of COX8A in PLC/PRF/5 and Hep3B cells was determined by qPCR and Western blot. **e** The *O*-GlcNAcylation levels on Rab3A in PLC/PRF/5 cells with indicated treatments. **f** mRNA and protein levels of COX8A in Hep3B cells with OGT knockdwon and/or Rab3A overexpression. **g** The *O*-GlcNAcylation levels on Rab3A in Hep3B cells with indicated treatments.
